# Transcranial Direct Current Stimulation Over the Primary and Secondary Somatosensory Cortices Transiently Improves Tactile Spatial Discrimination in Stroke Patients

**DOI:** 10.3389/fnins.2016.00128

**Published:** 2016-03-31

**Authors:** Shuhei Fujimoto, Noriko Kon, Yohei Otaka, Tomofumi Yamaguchi, Takeo Nakayama, Kunitsugu Kondo, Patrick Ragert, Satoshi Tanaka

**Affiliations:** ^1^Tokyo Bay Rehabilitation HospitalChiba, Japan; ^2^Laboratory of Psychology, Hamamatsu University School of MedicineShizuoka, Japan; ^3^Department of Public Health, Kyoto University Graduate School of MedicineKyoto, Japan; ^4^Medley, Inc.Tokyo, Japan; ^5^Department of Rehabilitation Medicine, Keio University School of MedicineTokyo, Japan; ^6^Department of Neurology, Max Planck Institute for Human Cognitive and Brain SciencesLeipzig, Germany; ^7^Faculty of Sport Science, Institute for General Kinesiology and Exercise Science, University of LeipzigLeipzig, Germany

**Keywords:** cortical plasticity, inter-hemispheric inhibition (IHI), palsy, grating orientation, transcranial direct current stimulation (tDCS), transcranial magnetic stimulation (TMS)

## Abstract

In healthy subjects, dual hemisphere transcranial direct current stimulation (tDCS) over the primary (S1) and secondary somatosensory cortices (S2) has been found to transiently enhance tactile performance. However, the effect of dual hemisphere tDCS on tactile performance in stroke patients with sensory deficits remains unknown. The purpose of this study was to investigate whether dual hemisphere tDCS over S1 and S2 could enhance tactile discrimination in stroke patients. We employed a double-blind, crossover, sham-controlled experimental design. Eight chronic stroke patients with sensory deficits participated in this study. We used a grating orientation task (GOT) to measure the tactile discriminative threshold of the affected and non-affected index fingers before, during, and 10 min after four tDCS conditions. For both the S1 and S2 conditions, we placed an anodal electrode over the lesioned hemisphere and a cathodal electrode over the opposite hemisphere. We applied tDCS at an intensity of 2 mA for 15 min in both S1 and S2 conditions. We included two sham conditions in which the positions of the electrodes and the current intensity were identical to that in the S1 and S2 conditions except that current was delivered for the initial 15 s only. We found that GOT thresholds for the affected index finger during and 10 min after the S1 and S2 conditions were significantly lower compared with each sham condition. GOT thresholds were not significantly different between the S1 and S2 conditions at any time point. We concluded that dual-hemisphere tDCS over S1 and S2 can transiently enhance tactile discriminative task performance in chronic stroke patients with sensory dysfunction.

## Introduction

Transcranial direct current stimulation (tDCS) is a process by which a weak direct current is passed through the skull, stimulating specific brain regions (Priori et al., 1998; Nitsche and Paulus, 2000, 2001; Furubayashi et al., 2008; Tatemoto et al., 2013). In stroke patients, tDCS over primary motor cortex (M1) has been found to improve motor performance in the affected upper/lower extremity (Tanaka et al., 2011; Elsner et al., 2013; Lüdemann-Podubecká et al., 2014; Pollock et al., 2014; Kang et al., 2015), as well as heighten muscle strength, motor learning, gait, and activities of daily living. Furthermore, tDCS has led to improvements in language and other cognitive functions in stroke patients (Elsner et al., 2013, 2015; Otal et al., 2015; Yang et al., 2015). However, the effect of tDCS on sensory dysfunctions in stroke patients remains unknown.

Previous studies have shown that tDCS can modulate somatosensory evoked potentials (SEP) and somatosensory processing (Matsunaga et al., 2004; Dieckhöfer et al., 2006; Boggio et al., 2008; Song et al., 2011; Costa et al., 2015; Sugawara et al., 2015; Wang et al., 2015; Nakagawa et al., 2016). For example, anodal tDCS over M1 led to increased SEP amplitude (Matsunaga et al., 2004), whereas cathodal tDCS over the primary somatosensory cortex (S1) led to decreased SEP amplitude (Dieckhöfer et al., 2006). Behaviorally, cathodal tDCS over S1 decreased participant performance on a tactile frequency discrimination task (Rogalewski et al., 2004), while anodal tDCS over S1 improved tactile spatial discrimination task performance (Ragert et al., 2008). A recent study reported that the repeated application of tDCS over S1 improved spatial tactile sensation in individuals with multiple sclerosis (MS) (Mori et al., 2012). These findings imply that tDCS may modulate somatosensory function, making it a potentially useful treatment for patients with somatosensory dysfunction (Song et al., 2011).

Dual-hemisphere tDCS, in which one hemisphere is excited while the other is inhibited, can have a powerful effect on behavioral performance (Vines et al., 2008; Kasahara et al., 2013; Fujimoto et al., 2014a; Sakai et al., 2014; Koyama et al., 2015). This improved performance appears to be the combined consequence of increased excitability in one hemisphere and decreased excitability in the other, likely via interhemispheric connections. There is some evidence of interhemispheric interactions between S1 and S2 in humans (Hoechstetter et al., 2001; Stancak et al., 2002; Werhahn et al., 2002; Hlushchuk and Hari, 2006; Ragert et al., 2011). We recently reported that, compared with single-hemisphere tDCS, a single session of dual-hemisphere tDCS over the primary somatosensory area (S1) (Fujimoto et al., 2014a) and secondary somatosensory area (S2) (Fujimoto et al., 2014b) transiently improved tactile discriminative performance in healthy subjects. Given these results, dual-hemisphere tDCS over S1 and/or S2 might improve somatosensory function in stroke patients with sensory deficits.

We used a double-blind crossover sham controlled study design to test two hypotheses. The first was that somatosensory performance in stroke patients with sensory dysfunction would be transiently enhanced by a single session of dual-hemisphere tDCS over S1 and S2, compared with sham stimulation. The second was that suppression of excitability in the un-affected hemisphere via cathodal tDCS would further increase excitability in the affected hemisphere, thus enhancing somatosensory performance in stroke patients.

## Materials and methods

### Patients

**Figure 1:**
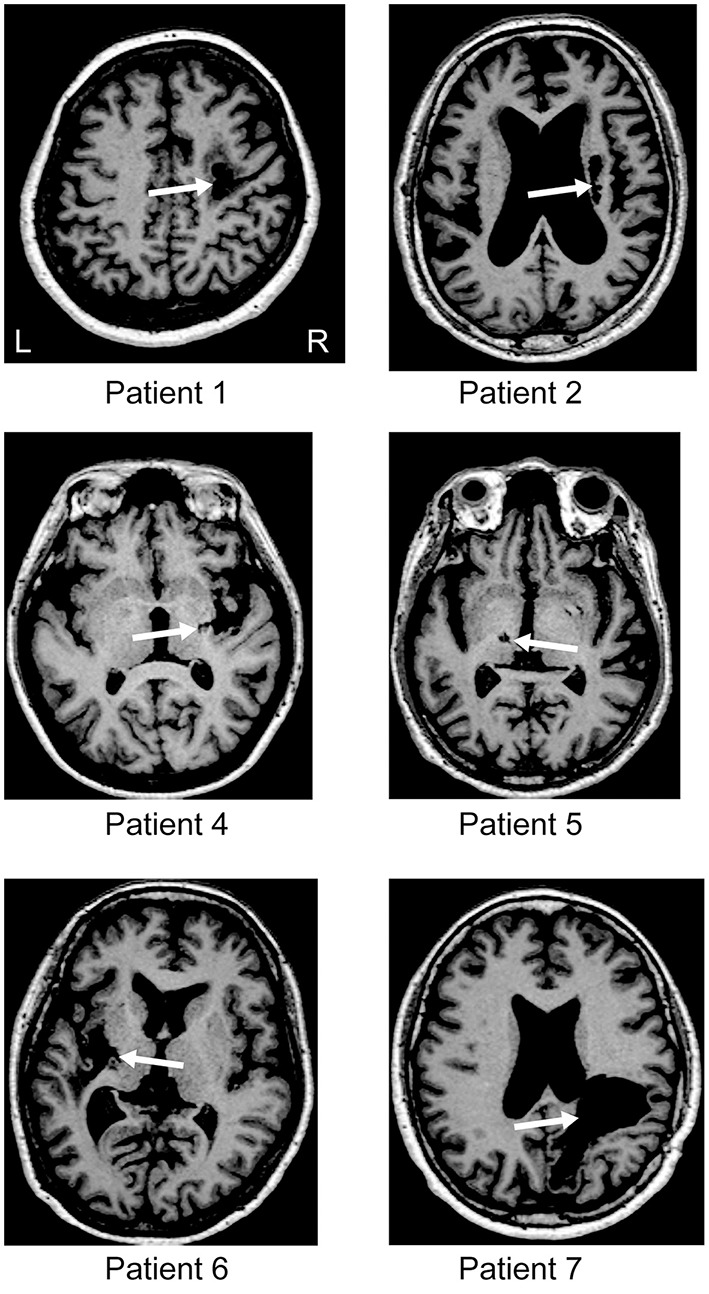
**Brain imaging**. T1 magnetic resonance imaging (MRI) at the level of the main stroke for each patient. For patients 3 and 8, the MRI data were missing. White arrows indicate the location of the lesion. L and R represent the left and right hemisphere, respectively.

Ten patients with chronic stroke participated in this study. However, according to a reviewer's comment, two patients' data (lesions in the brainstem or internal capsule) were excluded from the analysis in order to make the sample more homogenous. Thus, eight patients data (3 males and 5 females; mean age = 61.6 ± 9.0 years) were presented in the present article (Figure 1 and Table 1). It should be noted that even if the two excluded patients' data were added in the analysis, significant effect of tDCS was still observed, supporting our hypothesis. Participants met the following inclusion criteria: they had suffered a supratentorial stroke, they exhibited sensory deficits (excluding complete anesthesia), and they had obtained a Mini Mental Status Examination score of >24 points (Folstein et al., 1975). All participants were right hand dominant according to the Edinburgh Handedness Inventory (Oldfield, 1971), and none had a history of psychiatric or neurological illness. In the present study, participants were defined as having a sensory deficit if they exhibited impaired performance on at least one measure of sensory function via the stroke impairment assessment set (SIAS) (Touch or Position) (Chino et al., 1996), sensory function component of the Fugl-Meyer Assessment (FMA) (touch and position) (Sanford et al., 1993), or the Semmes-Weinstein monofilaments (SWM) exam (Semmes et al., 1960). All participants gave written informed consent before the experiments, which were approved by the local ethics committee at the Tokyo Bay Rehabilitation Hospital (No. 68-3).

**Table 1:** **Patient Information**.

**Characteristics**	**Patient No**.								**Mean ± SD**
	**1**	**2**	**3**	**4**	**5**	**6**	**7**	**8**	
Age, year	58	74	64	46	66	58	56	71	61.6 ± 9.0
Gender	F	M	M	F	M	F	F	F	
Time after stroke, month	60	47	89	49	10	55	49	105	58.0 ± 28.7
Lesion site	R corona radiata	R corona radiata	R putamen	R putamen	L thalamus	L putamen	R subcortex of parietal lobe	R putamen	
MMSE	26	27	30	27	30	30	30	27	28.4 ± 1.8
Handedness, EDS	R	R	R	R	R	R	R	R	
**SIAS MOTOR FUNCITON, U/E**									
Knee mouth test	3	3	3	3	3	3	2	1	2.6 ± 0.7
Finger function test	2	3	1a	1a	3	1b	1c	0	2.0 ± 1.4
SIAS SENSORY FUNCTION^*^, U/E									
Touch	2	2	2	2	1	1	1	1	1.5 ± 0.5
Position	1	2	3	2	2	1	1	2	1.8 ± 0.7
FMA SENSORY FUNCTION^**^, U/E									
Light touch(palm)	1	1	1	1	1	1	1	1	1.0 (all)
Position(thumb)	1	1	2	2	1	1	1	1	1.3 ± 0.5
SWM^***^	5	5	5	3	1	1	3	1	3.0 ± 1.9

*F, Female; M, Male; R, Right; L, Left; MMSE, Mini Mental State Examination; EDS, Edinburgh Handedness Scale; SIAS, Stroke Impairment Assessment Set; U/E, Upper extremity; FMA, Fugl-Meyer Assessment; SWM, Semmes Weinstein Monofilament*.

* *The SIAS is a comprehensive instrument that assesses sensory and motor function in stroke patients on a sensory scale of 0–3, where 0 = complete paralysis, 1 = severe paralysis, 2 = moderate paralysis, 3 = no paralysis, and a motor scale of 0–5, where 0 = complete paralysis, 1 = severe paralysis, 2 = moderate to severe paralysis, 3 = light to moderate paralysis, 4 = light to no paralysis, 5 = no paralysis*.

** *The FMA is a comprehensive instrument that assesses sensory function in stroke patients on a scale of 0–2, where 0 = anesthesia, 1 = hypoesthesia or dysesthesia, 2 = normal*.

*** *The Semmes-Weinstein Monofilament Test is an instrument that assesses a light touch function with various narrow monofilaments (2.83, 3.61, 4.31, 4.56, 6.65 Fmg). We counted 2.83, 3.61, 4.31, 4.56, 6.65 Fmg for 5, 4, 3, 2, 1 point*.

*1a: Minimal voluntary movement or mass flexion*.

*1b: Mass extension*.

*1c: Minimal individual movement*.

### Experimental procedure

We employed a double-blind, crossover, sham-controlled experimental design (Hummel et al., 2005; Gandiga et al., 2006). We measured performance of both index fingers in the grating orientation task (GOT) (Johnson and Phillips, 1981; Van Boven and Johnson, 1994; Nitsche et al., 2003) before, during, and after dual-hemisphere tDCS over S1 or S2, and before, during, and after sham tDCS over S1 or S2. We chose S1 and S2 as target regions because several previous studies have indicated that performance on the GOT task involves both S1 and S2 (Zhang et al., 2005; Fujimoto et al., 2014a,b).

All participants were exposed to 4 conditions (dual-hemisphere S1 tDCS, dual-hemisphere S2 tDCS, and the equivalent sham conditions for both regions), which were conducted in separate sessions at least 3 days apart. In the dual-hemisphere tDCS condition, anodal tDCS was applied over the lesioned hemisphere and cathodal tDCS was applied over the other hemisphere. In the sham condition, tDCS was applied over both hemispheres as in the experimental condition, but for only the first 15 s of the session.

The order of the four conditions was counterbalanced among the participants. Both the participants and the experimenter who measured GOT performance were blind regarding which sessions involved actual vs. sham stimulation. However, the experimenter could discern the S1 from S2 sessions because of the different electrode configurations. Before commencing the first session, the participants were familiarized with the tasks. Each session consisted of 3 task blocks (before, during, and 10 min after the intervention). After each session, we collected verbal rating scale (VRS) scores measuring the attention, fatigue, pain, and discomfort levels of the participants.

### Task

We evaluated spatial tactile discrimination performance using the GOT (Van Boven and Johnson, 1994). The GOT is a commonly accepted measure of tactile spatial acuity (Johnson and Phillips, 1981; Van Boven and Johnson, 1994). Additionally, a previous study reported that anodal tDCS over S1 had a facilitative effect on GOT performance (Ragert et al., 2008; Mori et al., 2012). During the task, participants sat on a chair in a comfortable position with their eyes covered by a blindfold. The tactile stimuli were applied using five hemispherical plastic domes with grooves (1.0, 1.2, 1.5, 2, 3, 4, 6, 8 mm in width) cut into their surfaces (Tactile Acuity Grating, Miyuki Giken). Using moderate force, the domes were applied onto the palmar side of the affected and non-affected index fingers for 2 s. The tests were performed separately for each index finger and the test order was counterbalanced among subjects. In each trial, the grooves of the dome were randomly applied in one of two directions: parallel or orthogonal to the axis of the index finger. Immediately after touching the domes, participants were expected to respond verbally, in a two-alternative force-choice paradigm, about whether the orientation of the grating of the presented dome had been parallel or orthogonal. Each dome was presented 20 times in one block (10 trials for the parallel and 10 trials for the orthogonal direction). In each block, the trials started with the largest grating (8.0 mm) and ended with the smallest grating (1.0 mm). To standardize the above procedures, we used a custom-made device that enabled the investigator to control the up-down movements of the domes. To minimize possible performance variance, a single trained investigator tested all of the participants. Using the percentage of correct grating discrimination responses, we calculated the threshold of accurate orientation detection as a primary outcome measurement. The threshold was calculated according to the following formula (Ragert et al., 2008):

(1) $$\begin{array}{l} \text{Threshold}={\text{G}}_{\text{below}}+(0.75-{\text{P}}_{\text{below}})/({\text{P}}_{\text{above}}-{\text{P}}_{\text{below}})\\ \;\times ({\text{G}}_{\text{above}}-{\text{G}}_{\text{below}}) \end{array}$$

G_below_: the grating spacing for which the subjects answered correctly in less than 75% of the trials

G_above_: the grating spacing for which the subjects answered correctly in more than 75% of the trials

P_below_: the percentage of correct responses for G_below_

P_above_: the percentage of correct responses for G_above_.

### Transcranial direct current stimulation

We applied tDCS using a DC Stimulator Plus (NeuroConn, Germany), which delivered direct current through two sponge surface electrodes (each with a surface area of 25 cm^2^). The intensity of the stimulation was 2 mA. In the dual-hemisphere tDCS condition, direct current was applied for 15 min (including the initial 15 s during which the current was gradually increased from 0 and the last 15 s during which it was gradually decreased to 0). The current density at the stimulation electrodes was 0.025 mA/cm^2^. These parameters are in accordance with previously published safety criteria and are far below the threshold for tissue damage (Nitsche et al., 2003; Poreisz et al., 2007). We used the same procedure in the sham condition, except that we applied current for only 15 s (Gandiga et al., 2006).

To identify the regions over the S1 and S2, we obtained T1-weighted images from all participants using magnetic resonance imaging (Philips, Intera 1.5T, Netherlands) before the tDCS experiment. For each participant, the centers of the stimulation electrodes were placed over the S1 and S2 regions that had been identified in the individual T1-weighted image. These areas were localized using a frameless stereotaxic navigation system (Brainsight2, Rogue Research Inc., Montreal, Canada). Mean Monteal Neurological Institute (MNI) coordinates for the center of the targeted locations across participants are follows: left S1 (x,y,z) = (−31.0 ± 2.1, −34.3 ± 3.9, 59.7 ± 2.7); right S1 = (34.0 ± 3.6, −33.0 ± 4.1, 58.7 ± 2.); left S2 = (−42.7 ± 1.6, −32.3 ± 2.0, 14.3 ± 2.3); right S2 (44.0 ± 2.2, −29.7 ± 2.0, 16.3 ± 1.5). Mean coordinates were calculated by means of anatomical normalization based on MNI coordinate system (Friston et al., 1995).

### Verbal rating scale

To address the possibility that the subjective state of the participants might influence their performance, we asked them to complete questionnaires in which they used a four-point scale to rate their levels of attention (1 = no distraction, 4 = highest level of distraction), fatigue (1 = no fatigue, 4 = highest level of fatigue), pain (1 = no pain, 4 = strongest pain), and discomfort (1 = no discomfort, 4 = strongest discomfort) at the end of each intervention (Poreisz et al., 2007).

### Statistical analysis

First, we separately analyzed the effects of the S1 and S2 stimulation compared with each respective sham condition. For the S1 stimulation condition, we calculated the GOT threshold for each participant in each block, and then subjected the threshold to a three-way repeated measures analysis of variance (ANOVA) with INTERVENTION (dual-hemisphere S1 or sham stimulation), TIME (pre, during, or 10 min after the intervention) and HAND (paretic or non-paretic hands) as within-subject factors. We adopted Bonferroni's test (two-tailed) for multiple-planned comparisons. We then repeated the same procedure for the S2 stimulation condition.

Second, we used a paired *t*-test to directly compare the mean GOT thresholds for the affected index finger during and 10 min after the real S1 and S2 simulations.

Finally, we analyzed the VRS scores using Fisher's exact test. For all statistics in the present study, the level of significance was defined as *p* < 0.05.

## Results

Individual data of GOT thresholds was shown in Table 2.

**Table 2:** **Individual data of grating orientation task (GOT) thresholds (mm)**.

**Case**	**S1 Sham**			**S1 Dual**			**S2 Sham**			**S2 Dual**		
	**Pre**	**During**	**Post 10 min**	**Pre**	**During**	**Post 10 min**	**Pre**	**During**	**Post 10 min**	**Pre**	**During**	**Post 10 min**
**AFFECTED INDEX FINGER**												
1	2.17	2.29	2.17	2.38	1.15	1.35	2.00	2.17	2.20	2.17	1.14	1.20
2	7.50	8.00	8.00	7.67	5.33	5.00	7.67	8.00	8.00	8.00	5.00	5.33
3	6.00	6.00	6.00	5.50	2.29	2.20	5.00	4.67	5.33	6.00	2.50	3.00
4	8.00	8.00	7.50	7.00	2.25	3.25	7.50	7.67	7.67	7.00	2.83	3.50
5	8.00	8.00	8.00	8.00	7.00	8.00	8.00	8.00	8.00	8.00	8.00	8.00
6	7.75	7.43	7.71	8.00	6.50	5.00	8.00	7.67	8.00	8.00	7.11	7.00
7	7.33	8.00	8.00	8.00	6.00	5.20	8.00	8.00	7.67	7.50	5.00	4.80
8	8.00	7.50	8.00	7.50	5.50	7.50	8.00	8.00	7.60	7.67	3.33	6.50
mean	6.84	6.90	6.92	6.76	4.50	4.69	6.77	6.77	6.81	6.79	4.37	4.92
SD	2.00	1.98	2.04	1.96	2.25	2.35	2.18	2.18	2.06	1.99	2.36	2.27
**NON-AFFECTED INDEX FINGER**												
1	1.20	1.20	1.10	1.28	1.20	1.10	1.20	1.17	1.20	1.13	1.20	1.13
2	2.00	2.20	2.25	2.20	2.00	2.25	2.00	2.20	2.00	2.00	2.00	2.00
3	4.00	3.50	4.00	3.67	3.20	2.25	4.00	3.50	3.80	3.80	3.63	3.83
4	1.35	1.40	1.35	1.38	1.35	1.25	1.37	1.25	1.30	1.45	1.50	1.45
5	4.50	4.00	3.86	4.00	3.25	3.33	3.43	3.75	3.63	3.63	3.63	3.60
6	2.25	2.33	2.40	2.25	2.17	1.25	2.50	2.67	2.50	3.50	3.00	2.50
7	1.43	1.50	1.44	1.50	1.33	1.26	1.50	1.46	1.50	1.43	1.50	1.50
8	2.50	2.33	2.50	2.50	2.50	2.67	8.00	7.00	7.67	2.71	2.67	2.83
mean	2.40	2.31	2.36	2.35	2.12	1.92	3.00	2.87	2.95	2.46	2.39	2.36
SD	1.23	1.00	1.10	1.02	0.82	0.83	2.25	1.94	2.15	1.09	0.97	1.01

### Effects of tDCS over S1

The three-way repeated measures ANOVA revealed significant main effects of INTERVENTION [*F*_(1, 7)_ = 18.71, *p* < 0.01], HAND [*F*_(1, 7)_ = 35.32, *p* < 0.01], and TIME [*F*_(2, 14)_ = 14.76, *p* < 0.01]. Additionally, the three-way interaction among INTERVENTION, HAND, and TIME was significant [*F*_(2, 14)_ = 14.53, *p* < 0.01]. This suggests that the real S1 and sham interventions had different effects on the GOT threshold between the paretic and non-paretic hand. To further explore this interaction, we performed a two-way repeated measures ANOVA for each hand.

**Figure 2:**
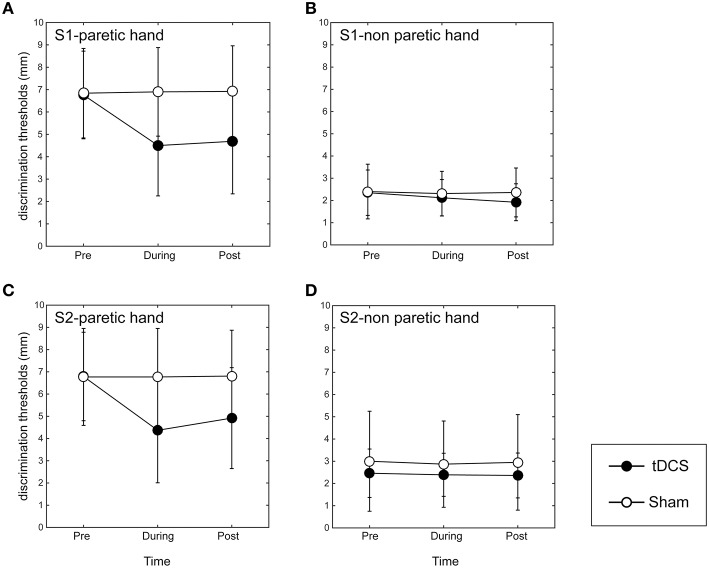
**Results of grating orientation task in dual-hemisphere S1 and S2 tDCS**. The mean threshold is plotted as a time course relative to the intervention, with bars indicating standard deviation (SD). **(A)** Indicates the effect of the stimulation on the affected index finger when adopting tDCS over S1. **(B)** Indicates the effect of the stimulation on the non-affected index finger when adopting tDCS over S1. **(C)** Indicates the effect of the stimulation over the affected index finger when adopting tDCS over S2. **(D)** Indicates the effect of the stimulation on the non-affected index finger when adopting tDCS over S2. Compared with sham tDCS (white circle, *p* < 0.05), dual-hemisphere tDCS (black circle) significantly improved the grating orientation threshold for the affected index finger during and 10 min after the stimulation over both S1 and S2. However, we found no significant effects of tDCS on the non-affected index finger, regardless of stimulation site.

In the paretic hand, the two-way repeated measures ANOVA revealed significant main effects of INTERVENTION [*F*_(1, 7)_ = 15.89, *p* < 0.01], TIME [*F*_(2, 14)_ = 13.17, *p* < 0.01], and their interaction [*F*_(2, 14)_ = 14.90, *p* < 0.01; Figure 2A]. A *post-hoc* analysis revealed that the GOT threshold during tDCS over S1 was significantly lower than that in the sham condition (*p* < 0.01). Additionally, 10 min after tDCS over S1, the GOT threshold was still significantly lower than that after the sham stimulation (*p* < 0.01).

On the non-affected index finger, the main effects of INTERVENTION [*F*_(1, 7)_ = 3.69], TIME [*F*_(2, 14)_ = 3.36], their interaction [*F*_(2, 14)_ = 2.53] were not significant (Figure 2B).

### Effects of tDCS over S2

The three-way repeated measures ANOVA revealed significant main effects of INTERVENTION [*F*_(1, 7)_ = 6.22, *p* < 0.05], HAND [*F*_(1, 7)_ = 22.41, *p* < 0.01], and TIME [*F*_(2, 14)_ = 12.60, *p* < 0.01]. Additionally, the three-way interaction among INTERVENTION, HAND, and TIME was significant [*F*_(2, 14)_ = 9.32, *p* < 0.01]. This suggests that the real S2 and sham interventions had different effects on the GOT threshold between the paretic and non-paretic hand. To further explore this interaction, we conducted a two-way repeated measures ANOVA for each hand.

In the paretic hand, the two-way repeated ANOVA revealed significant main effects of INTERVENTION [*F*_(1, 7)_ = 15.01, *p* < 0.01], TIME [*F*_(2, 14)_ = 15.40, *p* < 0.01], and their interaction [*F*_(2, 14)_ = 12.57, *p* < 0.01; Figure 2C]. A *post-hoc* analysis revealed that the GOT threshold during tDCS over S2 was significantly lower than in the sham condition (*p* < 0.01). Additionally, the GOT threshold 10 min after tDCS over S1 was still significantly lower than that after the sham stimulation (*p* < 0.01).

On the non-affected index finger, the main effects of INTERVENTION [*F*_(1, 7)_ = 0.78], TIME [*F*_(2, 14)_ = 0.88] and their interaction [*F*_(2, 14)_ = 0.32] were not significant (Figure 2D). These data indicate that there were no significant differences in GOT thresholds between the real S2 and sham stimulation on the non-affected index finger.

### GOT thresholds in S1 and S2 stimulation conditions

We compared the mean GOT thresholds for the affected index finger during and 10 min after the S1 and S2 tDCS stimulation sessions. We found no significant differences in the mean GOT thresholds between the S1 and S2 stimulations during [mean threshold of S1 stimulation = 4.50 ± 2.25; mean threshold of S2 stimulation = 4.37 ± 2.36; *t*_(7)_ = 0.38, *p* = 0.72] or 10 min after the intervention [mean threshold of S1 stimulation = 4.69 ± 2.35; mean threshold of S2 stimulation = 4.92 ± 2.27; *t*_(7)_ = 0.73, *p* = 0.49]. These results suggest that the GOT thresholds were not statistically different between the S1 and S2 stimulation sites.

### Psychological data

None of the participants reported side effects. The VRS scores recorded after each intervention revealed that the tDCS did not significantly influence participant levels of attention, fatigue, pain, or discomfort (Table 3). Thus, we expect that the confounding effects of these factors are minimal in this study.

**Table 3:** **Questionnaire scores after each intervention**.

	**Dual S1**	**Sham S1**	**Dual S2**	**Sham S2**	**Statistics (Fisher's exact test)**
Attention	1.13 ± 0.35	1.00	1.00	1.00	Non-significant
Fatigue	1.00	1.00	1.00	1.00	Non-significant
Pain	1.13 ± 0.35	1.00	1.13 ± 0.35	1.00	Non-significant
Discomfort	1.38 ± 0.52	1.00	1.25 ± 0.46	1.13 ± 0.35	Non-significant

*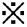 1.00:All of participants points 1. Data represent the group mean ± SD. Attention was scored on a scale of 1–4 (1 = no distraction; 4 = highest level of distraction). Fatigue was scored on a scale of 1–4 (1 = no fatigue; 4 = highest level of fatigue). Pain was scored on a scale of 1–4 (1 = no pain; 4 = strongest pain). Discomfort was scored on a scale of 1–4 (1 = no discomfort; 4 = strongest discomfort)*.

## Discussion

Our results indicate that both dual-hemisphere S1 and S2 tDCS transiently improved tactile spatial discrimination task performance compared with sham stimulation in stroke patients with sensory deficits. This effect was specific to the affected index finger. The effecter-specificity of the modulation indicated that general effects, such as changes in attention, fatigue, or pain/discomfort, did not cause the results. The VRS scores supported this notion.

Previous studies with stroke patients have reported that tDCS can enhance motor (Hummel et al., 2005; Hummel and Cohen, 2006; Tanaka et al., 2011), language, and cognitive functions (Shah et al., 2013; Flöel, 2014). Regarding sensory deficits, previous studies have reported on the therapeutic effect of tDCS in patients with multiple sclerosis, peripheral nerve neuropathic pain, and tinnitus (Mori et al., 2012; Nizard et al., 2012; Song et al., 2012). However, to the best of our knowledge, the present study is the first to show that tDCS can improve somatosensory function in stroke patients with sensory deficits.

Our finding that dual-hemisphere tDCS over S1 and S2 improved the GOT threshold in stroke patients is consistent with the findings of a previous study that showed that dual-hemisphere tDCS over the bilateral S1 and S2 enhanced GOT performance in healthy subjects (Fujimoto et al., 2014a,b). In our dual-hemisphere tDCS protocol, the anodal tDCS might have increased the excitability of the affected hemisphere, thus affecting tactile spatial discrimination in the affected index finger. Concurrently, decreased excitability in un-affected hemisphere induced by cathodal tDCS might have further increased excitability in the affected hemisphere through a reduction in interhemispheric inhibition (Werhahn et al., 2002; Hlushchuk and Hari, 2006; Ragert et al., 2011). We speculate that the combined effect of increased excitability in the affected hemisphere via anodal tDCS and decreased excitability in the un-affected hemisphere via cathodal tDCS might have led to the observed behavioral gain.

In the present study, we exclusively used dual-hemisphere tDCS. Thus, we cannot rule out the possibility that single-hemisphere tDCS might have been sufficient to improve tactile discrimination performance in our sample of stroke patients. However, in previous experiments with healthy subjects, dual-hemisphere tDCS elicited a more robust improvement in performance compared with single-hemisphere tDCS (Fujimoto et al., 2014a,b). Therefore, it is reasonable to expect that dual-hemisphere tDCS represents a more powerful strategy for improving tactile spatial discrimination performance in stroke patients compared with single-hemisphere tDCS (Vines et al., 2008; Kasahara et al., 2013; Fujimoto et al., 2014a; Sakai et al., 2014; Koyama et al., 2015). Future studies could clarify this issue by investigating the effects of single-hemisphere stimulation on behavior.

In the present study, we found the degree of improvement in GOT performance elicited by S1 and S2 stimulation to be comparable. Therefore, we cannot make a judgment about which somatosensory cortex is a more suitable target for sensory improvement in stroke patients. This result is consistent with previous neuroimaging findings that both S1 and S2 are important for the performance of tactile spatial discrimination tasks (Zhang et al., 2005).

One limitation in the present study was that the patients were relatively heterogeneous in terms of stroke localization (corona radiate, putamen, thalamus, and subcortical region). Therefore, it is difficult to conclude whether the observed reduction in tactile threshold is due to a potentiation or improvement of an impaired sensory tract by tDCS. Stroke patients with more homogeneus pathology should be investigated in future. On the other hand, the heterogeneity in the present study could be strength when we consider the wider therapeutic impact of tDCS in real life.

To conclude, our study appears to be the first double-blind, cross-over, sham-controlled experiment to demonstrate that dual-hemisphere tDCS over S1 and S2 can enhance tactile spatial discrimination in chronic stroke patients with sensory deficits. Our results provide evidence for the efficacy of tDCS in improving somatosensory function after chronic stroke. Although our small number of subjects may limit the strength of our conclusion, our findings raise the possibility that repeated applications of tDCS, combined with rehabilitation training, might have a long-term beneficial effect on somatosensory performance in stoke patients, as shown with upper limb motor training (Reis et al., 2009; Lefebvre et al., 2012). Testing this hypothesis will be relevant to the clinical application of non-invasive cortical stimulation.

## Author contributions

ST conceived and supervised the study. SF, PR, ST designed the experiments. SF, NK, YO, TY, KK carried out the experiments. SF, NK, ST analyzed the data. SF, TN, PR, ST wrote the manuscript. All authors approved the final version of the submitted manuscript.

### Conflict of interest statement

The authors declare that the research was conducted in the absence of any commercial or financial relationships that could be construed as a potential conflict of interest.

## Abbreviations

FMA: Fugl-Meyer Assessment
GOT: Grating orientation task
SIAS: The stroke impairment assessment set
SWM: Semmes-Weuinstein monofilaments
S1: The primary somatosensory cortex
S2: The secondary somatosensory cortex
tDCS: Transcranial direct current stimulation
VRS: Verbal rating scale.
